# Systematic analysis of biological endpoint variability and implications for quantitative modeling of the FLASH sparing effect

**DOI:** 10.1016/j.phro.2026.100915

**Published:** 2026-02-02

**Authors:** Isabella Colizzi, Mathilde Toschini, Antony J. Lomax, Serena Psoroulas

**Affiliations:** a Center for Proton Therapy, Paul Scherrer Institut, 5232 Villigen, PSI, Switzerland; b Department of Physics, ETH Zurich 8092 Zurich, Switzerland; c Department of Radiation Oncology, University Hospital Zurich (USZ), University of Zurich (UZH), Raemistrasse 100, CH-8091 Zurich, Switzerland

**Keywords:** FLASH, FLASH effect, UHDR, Meta-analysis, Preclinical studies, Endpoints, Guidelines, Modelling

## Abstract

**Background and purpose:**

Ultra-high dose rate (UHDR) radiotherapy shows promise in sparing normal tissue while maintaining tumor control. However, heterogeneity across preclinical experiments limits cross-study comparability and the development of predictive models. By employing both qualitative and quantitative analyses, we aim to identify variability in endpoints, clarify inconsistencies, and highlight knowledge gaps critical for future quantitative modeling.

**Material and methods:**

We reviewed in-vivo proton and electron FLASH murine experiments (1966–2024) and extracted commonly reported endpoints. A subset of comparable endpoints—crypt cell ratio and survival (abdominal), maximum moist desquamation (skin), and Novel-Object-Recognition tests (brain)—was analyzed using qualitative comparison and logistic regression to assess parameters potentially driving the FLASH effect.

**Results:**

Fifty papers were reviewed, revealing substantial differences in endpoint selection, timing, and methodology. Most endpoints were not directly comparable across studies, with only four endpoints amenable to cross-study analysis. Logistic regression was constrained by high collinearity among parameters, reflecting both experimental heterogeneity and limited harmonization.

**Conclusion:**

Our review highlights the urgent need for standardization in biological endpoints and methodologies in FLASH research. By quantifying variability in preclinical FLASH data and identifying comparable endpoints, our work provides essential guidance for future modeling efforts, bridging biological evidence and quantitative approaches for treatment planning applications.

## Highlights

• Systematically reviews in vivo FLASH studies and quantifies endpoint variability.
• Identifies comparable endpoints suitable for cross-study analysis and modeling.
• Reveals key knowledge gaps and strategies to improve data comparability.

## Graphical abstract

**Figure:**
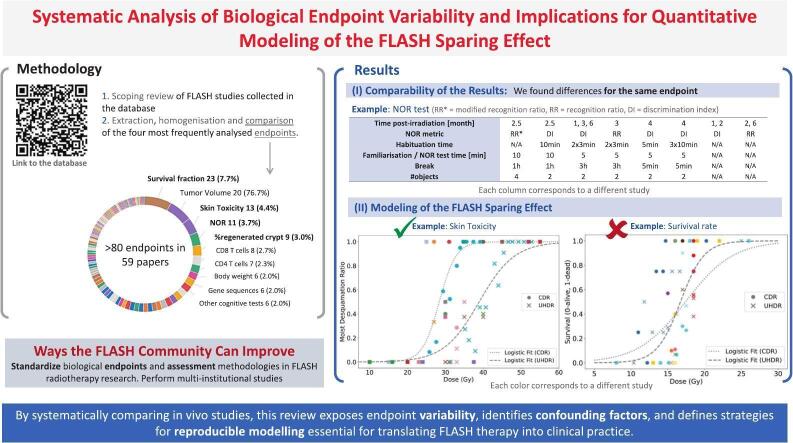

## 1 Introduction

The so-called ‘FLASH effect’ is an enhancement of normal tissue protection while concurrently eliciting an equivalent tumor response [1], [2]. This effect was observed across various beam modalities, species, organs, and endpoints at ultra-high dose rates (UHDR), defined as exceeding 40 Gy/s, compared to conventional dose rates (CDR) ranging from 0.03-2 Gy/s [3]. Ten years after the first use of the term’FLASH’ [4], our understanding of the parameters and conditions necessary to observe a FLASH effect remains incomplete. Although current biological findings already support a simplified clinical directive, our goal is to go beyond it. For the safe and effective clinical implementation of FLASH radiotherapy, robust models within treatment planning systems (TPS) are necessary [5], [6], [7]. This modeling should account for variations across different biological endpoints, differentiate between short- and long-term toxicities, consider organ-specific responses, and incorporate beam delivery parameters such as pulse structure and dose rate [8]. By integrating this type of modeling into TPS, as is done for biological modeling in ion therapies [9], [10] and for α/β ratios in conventional fractionation [11], we can enhance outcome predictions and effectively utilize the FLASH effect.

While most reviews have provided qualitative assessments [12], [13], [14], [15], [16], [17], recent studies began to incorporate quantitative analyses [18], [19], [20]. Building on these efforts, and recognizing the critical need to systematically evaluate and synthesize the available biological evidence [21], [22], we aimed to deliver a comprehensive, data-driven evaluation of the FLASH effect on healthy tissue. We extracted and analyzed biological endpoints from published studies, acknowledging that variability in experimental models and outcome measures necessitates a structured approach for meaningful comparisons. When feasible, we explored which factors were statistically related to differences between UHDR and CDR irradiation.

This scoping review provides a high-level overview of the FLASH sparing effect, systematically comparing biological outcomes, identifying areas and potential reasons for limited or inconsistent evidence, clarifying discrepancies in existing data, and highlighting key knowledge gaps that hinder modeling of the FLASH effect.

## 2 Materials and methods

### 2.1 Data collection and preparation

**Fig. 1:**
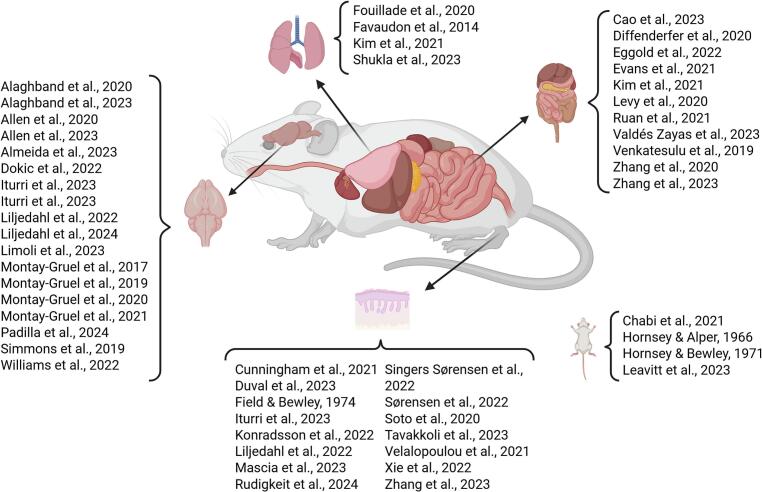
List of papers per irradiated areas. Created in BioRender. Colizzi, I. (2026) https://BioRender.com/p75b023.

A scoping review was conducted using a publicly available, web-based database [23] of in-vivo murine studies comparing UHDR and CDR irradiation, published between 1966 and 2024. This systematically compiled database contains key experimental parameters (e.g., dose rates, beam modalities), biological models, and endpoints (e.g., tumor control, normal tissue effects) to enable FLASH effect *meta*-analyses. It reviewed 59 articles restricted here to electrons (31) and protons (18, with 1 comparing both), the most common beam types. The literature was grouped into four primary irradiation sites, see Fig. 1: abdomen (11), skin (15), brain (18), lung (4), and whole body (4). More than 80 distinct endpoints were identified, of which only 29 were evaluated in more than two studies. The analysis focused on tissue-sparing effects, with key endpoints including survival fraction, crypt cell regeneration, skin toxicity, and the Novel-Object-Recognition (NOR) test. Experiments performed with tumor-bearing mice were excluded from survival assays because tumor presence would influence the endpoint.

### 2.2 Endpoint by endpoint comparison

The impact of CDR and UHDR irradiation was evaluated as a function of dose in different studies for the individualized endpoints. The definition of “CDR” and “UHDR” groups followed that provided in the corresponding publication. While we highlighted additional variables such as mouse strain, irradiated area, oxygenation status, and analysis type or timing when available and relevant for interpretation, we recognize that not all potentially influential parameters could be uniformly accounted for. This was not a matter of selective reporting, but a consequence of data availability across studies. As such, some parameters inevitably remained as potential confounding factors.

### 2.3 Statistical analysis

The open dataset [24], which provides information on biological and physical parameters for each irradiated mouse, was used, and the “FLASH effect” was parameterized as described in [23]. Because many experiments investigated only one dose level, we avoided using any FLASH-modifying factor in the analysis.

Ideally, all simple or derived variables that could enhance the model's predictive value should be tested. However, collinearity between parameters can compromise the robustness of results. We identified statistically independent variables using the Spearman [25] rank correlation coefficient (SRCC). Variables with an SRCC *>* 0.8 were considered to be cross-correlated. If so, the variable with the lower correlation with the effect was removed from the model. From this reduced set, model parameters were selected for inclusion in the logistic regression model using stepwise selection (*stepwiseglm* MATLAB function). Afterward, each model’s performance was evaluated using McFadden’s Pseudo R-squared test [26] and the ROC test. We performed a Leave-One-Group-Out Cross-Validation (LOGO-CV) to assess how well the model generalizes to unseen data by iterative training on n-1 groups (papers) and testing on the left-out group, and single-data and group-based bootstrapping to test model variability and confidence intervals.

## 3 Results

### 3.1 Data collection and endpoint by endpoint comparison

#### 3.1.1 Survival fraction

**Fig. 2:**
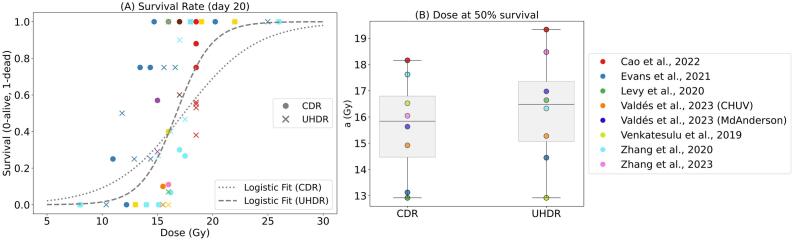
(A) Survival rate at day 20 as a function of dose for CDR and UHDR irradiated mice. A weighted logistic fit (1/(1 + e^(a−x)/b^) over all the data points is performed in both cases. The fitting parameters (a, b) with their 95% confidence intervals are (17.40 ± 0.67, 3.24 ± 1.05) for CDR and (16.72 ± 0.26, 1.61 ± 0.30) for UHDR. The compared papers are listed in Table S1. (B) Parameter a of the logistic fit of each single group. The data are grouped in CDR and UHDR. The box plot shows the quartiles of the distribution, and the horizontal line shows the median. Details of each fit are reported in Fig. S1 and Table S6.

Numerous research groups investigated survival fraction, with 20 articles [27], [28], [29], [30], [31], [32], [33], [34], [35], [36], [37], [38], [39], [40], [41], [42], [43], [44], [45], [46] exploring this endpoint (see Table S1). Fig. 2(A) reports the survival rate after abdominal irradiation of non-tumor-bearing mice on day 20, as all reviewed studies indicated no changes beyond this point. Hornsey et al. [45], [46] were excluded as they only reported the survival rate five days post-irradiation. The dose level at which the CDR and the UHDR irradiated groups separate varies between experiments, and no clear FLASH sparing effect can be identified when fitting to overall survival. By fitting each group separately, see Fig. 2(B) and Fig. S1, we observe that for two out of seven studies (Zhang et al. 2020 [29] and Venkatesulu et al. 2019 [35]) there is a decrease in survival at UHDR.

A critical factor in determining the survival fraction is the euthanasia criterion. This was not consistently reported across studies and, when reported, varied, ranging, for example, from 15% to 30% of maximum weight loss (see Supplementary A). Considering only weight loss as the euthanasia criterion, a threshold reduction from 20% (Valdès et al., 2023 [36] /MD Anderson) to 15% (Valdès et al., 2023 [36] /CHUV ) led to a decrease in the dose associated with the 50% survival rate, see Fig. 2(B). A similar consideration could explain the high rate observed in Cao et al. 2022 [32] (weight loss *>* 30%). Given this inconsistency and the frequent absence of precise values, we acknowledge this variability as a confounding factor. Additional confounding factors that could not be clearly separated in the qualitative analysis were the mouse strains (C57BL/6J for all studies excluding Venkatesulu et al. 2019 [35], which used BALB/c), the type of anesthesia (Ketalar/Rompun in most studies), and the irradiated area (mostly whole-abdominal electron irradiation). In Evans et al., 2021 [27], isoflurane gas was used together with a small focal proton beam (11  mm diameter); these differences may explain the low survival ratio observed in that study.

#### 3.1.2 Crypt cells

**Fig. 3:**
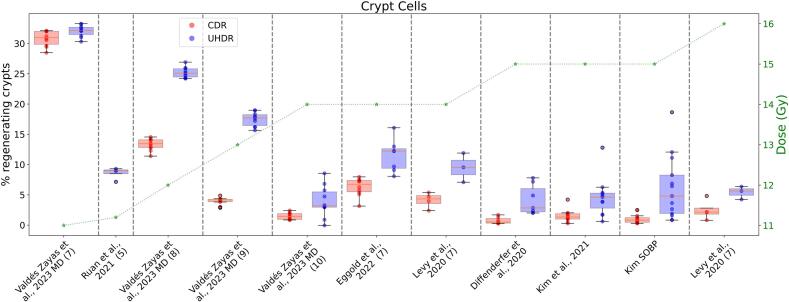
Percent of the regenerative crypt cells at 3.5-4d post-irradiation weighted for the irradiated area. For electron irradiation, the number of pulses used in each paper is reported in brackets (Ruan et al., 2021 [49] reports only data in UHDR for 5 pulses). The box plot shows the quartiles of the distribution, and the horizontal line shows the median. The compared papers are listed in Table S2.

**Fig. 4:**
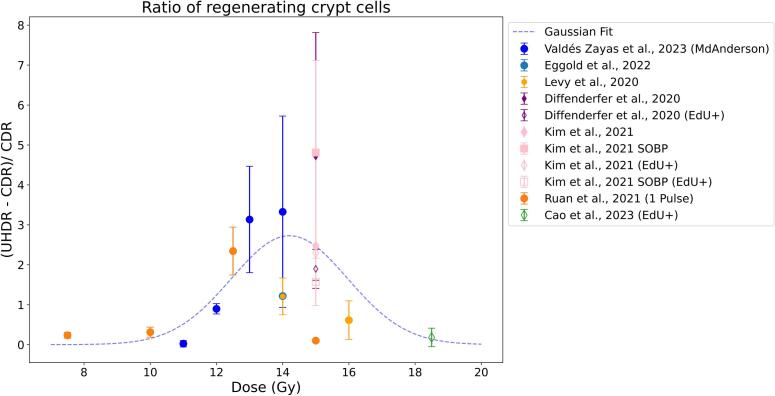
Relative difference of regenerated crypt cells and number of EdU + cells/crypt after irradiation. The dotted line is a Gaussian fit a·e^−((x−m)/s)2/2^ of the weighted data (excluding the data labeled with EdU + ). The fitting parameters (a, m, s) with their 95% confidence intervals are (2.72 ± 1.23, 14.21 ± 1.29, 1.75 ± 1.55). The compared papers are listed in Table S2.

Eight studies [28], [30], [33], [36], [47], [48], [49], [50] (see Table S2) investigated the number of crypt cells regenerated after irradiation as evidence of the FLASH effect on early side effects. Electron irradiation was usually performed with a field perpendicular to the abdominal region, whereas different orientations were used for proton irradiation. All studies used the C57BL/6J mouse model, except for Ruan et al. 2021 [49], which was the only study that also used oxygen-enhanced air (95% O2) during irradiation. We compared only data reporting the number of crypt cells at 3.5–4 days post-irradiation, as this is the window of turnover for crypt regeneration [36]. For electron irradiation, only data with a comparable number of pulses were considered (Fig. S2 includes the whole dataset). To enable comparison between studies reporting either percentages or absolute numbers of crypt cells, the latter was normalized assuming an average of 140 crypts per circumference (Fig. S3 evaluates the robustness of our assumption), as reported in the literature [51], [52], [53], [54], [55]. This approach enabled consistent interpretation across datasets and accounted for variations in the size of the irradiated area. Fig. 3 shows that the medians of the distributions for the same dose are comparable across different papers except for Ruan et al. 2021 [49], which may differ for reasons explained above. Further, Valdès Zayas et al., 2023 (10) [36] does not align with Eggold et al., 2022 [47] and Levy et al., 2020 [33], probably due to the different evaluation day (3.5d vs. 4d). Fig. 4 shows the relative difference between CDR and UHDR irradiation as a function of the prescribed dose. We observe a better dose–response between 12 and 16 Gy, as well as variability in the outcomes between the two evaluation methods used (EdU + and crypt cell counts).

#### 3.1.3 Moist desquamation

**Fig. 5:**
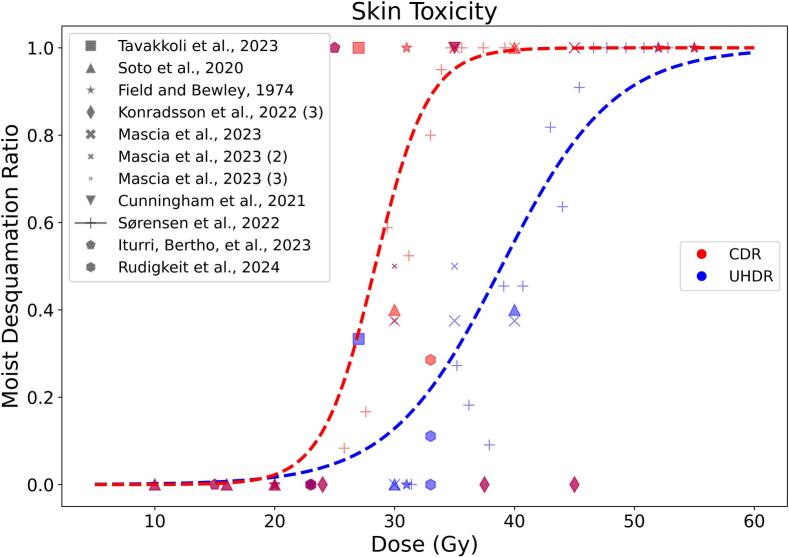
Percent of moist desquamation as a function of the total delivered dose (if fractionation was used, the number of fractions is reported in parentheses). The dotted line represents the weighted logistic fit (1/(1 + e^(a−x)/b^)) of all CDR and UHDR irradiated mice. The fitting parameters (a, b) with their 95% confidence intervals are (28.37 ± 0.35, 2.22 ± 0.28) for CDR and (38.93 ± 0.52, 4.67 ± 0.42) for UHDR. The compared papers are listed in Table S3 − Sørensen et al., 2022 [62] and Sørensen et al., 2022 [63] are considered as one single entry.

Another frequently analyzed endpoint is skin toxicity.^2^ Each research group used a different scoring system, which was converted into a harmonized classification based on three levels [23] − light toxicity, moist desquamation, and severe toxicity (the conversion was not possible for Tavakkoli et al., 2024 [56], which uses a binary scoring system). Moist desquamation can be easily identified, making it a meaningful endpoint for skin irradiation. Although the timing of the peak reaction does not correlate with the delivered dose, the maximum severity of toxicity clearly shows a dose-dependent relationship [57]. Therefore, we only focused on the percentage of mice reaching moist desquamation as a function of dose. The eleven papers [34], [43], [56], [58], [59], [60], [61], [62], [63], [64], [65] evaluating this endpoint are listed in Table S3. Fig. 5 shows the percentage of rodents (Fischer 344 rats in [43], [59], [64]) that reached moist desquamation after irradiation (under normoxic conditions) during the healing period. The logistic fit of each single group is available in the supplementary materials (Fig. S4–S5, Table S7). In the dose range 20–40  Gy, a clear distinction emerges between the CDR and UHDR irradiated groups. A notable confounding factor, often not reported, is skin preparation before irradiation [66]: more aggressive depilation can lead to a stronger skin reaction (see Cunningham et al., 2021 [61] compared with Mascia et al., 2023 [60] in Fig. S5).

#### 3.1.4 Novel object recognition

Brain irradiation is investigated in FLASH literature for long-term radiation effects. Various behavioral tests can assess cognitive function; the most common is the NOR, which was used in 11 studies [39], [64], [67], [68], [69], [70], [71], [72], [73], [74], [75] (see Table S4). Other tests, like the open field test, the light–dark box test, or objects in the updated location, have also been investigated, but in fewer studies. Overall, all articles except Williams et al. 2022 [75] reported improved behavioral and memory performance after UHDR irradiation. This exception might be due to the biological model, the very young age of the rat, and/or the low doses delivered.

**Fig. 6:**
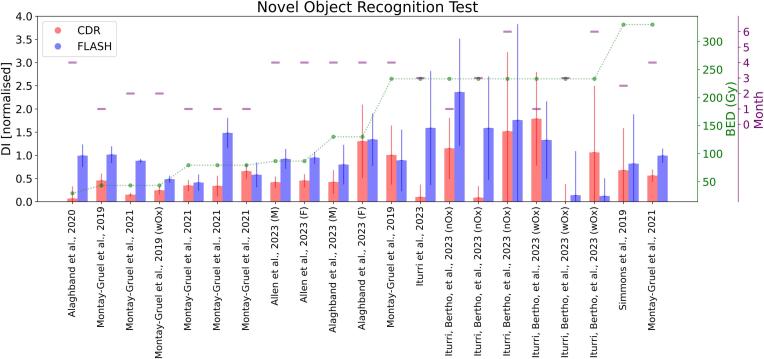
Novel-Object-Recognition test, DI is normalized for the control group. The compared papers are listed in Table S4.”nOx” indicates without Oxygen,”wOx”, with. M and F indicate the study’s exclusive use of male or female mice. In green is the BED (α/β = 3) at which the rodent was irradiated, and in purple is the month at which the NOR test was performed. (For interpretation of the references to colour in this figure legend, the reader is referred to the web version of this article.)

We compared only papers that report NOR test results as a discrimination index (DI), the most commonly used metric. Due to variability in the control group response (see Fig. S6), each paper's DI was normalized to the control group. Fig. 6 shows that, overall, the CDR-irradiated groups had worse outcomes (within the uncertainty range) compared to the UHDR groups, except for Iturri, Bertho et al., 2023 [64], where rodents received supplemental oxygen during anesthesia, and Alaghband et al., 2023 (F) [70] and Montay-Gruel et al., 2019 [68], where CDR performed slightly better than UHDR. Also, probably due to the different biological models used in the study, the DI reported in the two papers from the Institute Curie [64], [73] was higher than the others. The magnitude of the difference between CDR and UHDR is not consistent across studies, potentially due to variations in the time points of analysis or inherent variability of the NOR test protocol (see Supplementary A4). The use of single-dose levels in most experiments limited the ability to assess dose dependence or identify consistent patterns in the observed effects.

### 3.2 Statistical analysis

The only endpoints with a large comparable dataset were survival fraction, crypt cells, and moist desquamation. Supplementary C includes details of the statistical analysis.

#### 3.2.1 Survival fraction

Highly correlated variables (e.g., mouse age and LET or anesthesia and delivery time, see Fig. S7 for details) were removed from the modeling process to address collinearity. After logistic regression, the most significant parameters identified were associated with the experimental setup and the chosen biological model, such as the type of anesthesia, the rodents’ sex, and the irradiation volume. Of the beam delivery parameters, only the delivery time and the field dose rate in the UHDR regime appeared significant (see Table S8). While model performance was good, cross-validation and bootstrap resulted in poor generalization. We observed overfitting, and the model’s performance varied wildly depending on which groups were used for modeling/testing (see Fig. S8).

#### 3.2.2 Crypt cells

The logistic analysis showed only the intercept in the final model, meaning that the stepwise selection process did not find any statistically significant predictors.

#### 3.2.3 Moist desquamation

For the moist desquamation endpoint, the regression analysis on the non-cross-correlated variables (see Fig. S9-S10) identified several statistically significant parameters related to the experimental setup and the chosen biological model (see Table S9). These included the oxygen level during irradiation, the type of tissue irradiated (such as heart, foot, or leg), the method of hair removal (electric trimmer or cream, causing less skin inflammation), and the age of the mice. In addition, parameters related to the pulse structure of the beam, dose per pulse (positively correlated with dose), and spot duration (which equals the irradiation time for electron irradiation) in the UHDR regime were significant for modeling the effect. However, LOGO-CV and Bootstrap showed that the model was sensitive to specific groups, resulting in possible prediction errors (see Fig. S11).

## 4 Discussion

This study presented a scoping review of in vivo proton and electron FLASH research, aiming to systematically compare biological outcomes, identify limitations and conditions for comparison, and highlight knowledge gaps that hinder quantitative modeling, ultimately providing practical guidance for future research.

An important outcome of this study was the recognition of inconsistent biological endpoints and reporting standards across studies, which presents a major barrier to quantitative comparison and *meta*-analysis. We identified 80 distinct endpoints across 50 publications. However, very few endpoints, such as remaining crypt cells, survival rate, NOR test, and skin toxicity after irradiation, were investigated by multiple groups. Overall, early reactions like the crypt cell assay and moist desquamation demonstrated consistent results across the literature, suggesting an expansion of the therapeutic window for these endpoints UHDR. However, this result is less apparent for other endpoints. An overall sparing effect at UHDR was observed in the NOR test, although the extent of sparing varied across studies. Similarly, for the day-20 survival rate assays, the results varied widely, even within the CDR-irradiated group. Because this endpoint primarily reflects acute side effects, future studies should assess long-term survival to better capture late effects.

A critical challenge in interpreting preclinical outcomes across studies lies in the variability in how and when endpoints are defined and evaluated, a problem also present in multi-institutional studies [36]. Significant differences in assay types and metrics compound this inconsistency, greatly influencing the outcomes and comparability of results. The timing of post-irradiation analysis further complicates interpretation, as outcomes may vary depending on when assessments are performed. Moreover, the format in which data is reported, whether as percentages or absolute numbers, can significantly affect how findings are compared across studies. Supplementary A provides a detailed overview of how the different endpoints were reported across publications, highlighting these issues and facilitating future cross-study comparisons.

We conducted statistical modeling on three endpoints: survival rate, moist desquamation, and survival fraction following gut irradiation. Our analysis provides preliminary insights into the potential significance of certain variables, as also supported by other studies [20], [56], [60], [61], [76]. Although the model performs well, cross-validation and bootstrap reveal overfitting and group effects. Collinearity existed among parameters due to different research groups' unique setups and machine configurations. Future preclinical modeling should explicitly address collinearity and incorporate it into experimental design.

Despite inherent limitations, several recommendations emerge from the analysis. The diversity in endpoints and study designs underscores the need for standardized reporting and harmonized methodologies within the FLASH research community. While a proposal for standardizing physical parameters has been published [77], a similar effort for biological parameters would be advantageous. Existing guidelines for grading radiation-induced toxicities [78] face variability in interpretation, complicating reproducibility. Standardizing reporting and analysis of common endpoints will be an important step towards achieving consensus in the description of the FLASH effect. Ideally, it should be done within international societies; in Supplementary Material A (Table S5), we make a first proposal in this direction, based on our observations on the literature. Standardization will not hinder the exploration of biological parameters. Instead, it will help various groups report data consistently and comparably analyze the same endpoint. When standardization is not feasible, multi-institutional studies could investigate methods to compare different models.

For a successful clinical translation of FLASH radiotherapy, it is crucial to incorporate dose and dose rate thresholds [79], along with tissue-specific”dose-modifying factors” [2], [80] into the TPS. However, the current literature is unable to provide these data fully. To advance our understanding and improve the quantification of outcome variability, future studies should adopt a more systematic approach. This includes investigating dose (and dose rate) response relationships using multiple dose (and dose rate) levels and designing experiments that minimize correlations between physical and biological parameters. It is particularly important to avoid exclusively relying on single-dose measurements and include additional dose levels that produce intermediate response probabilities, enabling the data to support estimation of the side effect development curve and inflection point.

In conclusion, our study highlights the importance of standardizing biological endpoints and assessment methodologies in FLASH research to minimize variability and enhance the comparability of results. Collaborative efforts focused on experimental variations and transparent, standardized reporting are crucial for promoting reproducibility and facilitating robust modeling of the FLASH effect for future clinical applications.

## CRediT authorship contribution statement

**Isabella Colizzi:** Supervision, Data curation, Formal analysis, Methodology, Visualization, Conceptualization, Writing – original draft. **Mathilde Toschini:** Data curation, Formal analysis, Methodology, Visualization, Writing – original draft. **Antony J. Lomax:** Supervision, Funding acquisition, Project administration, Writing – review & editing. **Serena Psoroulas:** Supervision, Conceptualization, Funding acquisition, Project administration, Writing – review & editing.

## Declaration of competing interest

The authors declare that they have no known competing financial interests or personal relationships that could have appeared to influence the work reported in this paper.

## Footnotes

2 “skin toxicity” is used specifically in reference to preclinical murine studies, where the term denotes experimentally induced tissue response to high doses.
