# Supplementary material for: Systematic analysis of biological endpoint variability and implications for quantitative modeling of the FLASH sparing effect
Source: Phys Imaging Radiat Oncol. 2026 Feb 2;37:100915. doi: 10.1016/j.phro.2026.100915 (PMC12906097; doi:10.1016/j.phro.2026.100915)
Supplement: Supplementary Data 1 [file mmc1.pdf]

Supplementary A: Endpoint evaluation

This section presents additional information we have extracted from the papers to facilitate a comparative analysis of the different outcomes presented in the main article. As each group performs its type of analysis, it is essential to clearly understand the similarities and differences of every test when comparing outcomes from different centers.The descriptions in this section are mainly rephrased from the original papers. In addition, tables with all the analysed papers are reported.

A1. Survival (Euthanasia criteria)

When talking about survival fraction, we should understand that the euthanasia of the animal is undertaken when specific criteria are met, and the requirements are not always the same between different facilities. For this reason, we will report the euthanasia criteria in the following section, as reported in the papers. We will only consider the irradiation of non-tumor-bearing mice.

- Evans et al., 2021 [1]: Any mice that lost more than the 20% of the initial weight were euthanized
- Zhang et al., 2023 [2] and Zhang et al., 2020 [3]: not given
- Cao et al., 2022 [4]: Upon onset of severe morbidity including hunched posture, social withdrawal, relative immobility, or apparent weight loss > 30%
- Soto et al., 2020 [5]: Mice whose cutaneous lesions progressed despite treatment were humanely euthanized at, or before, the 8-week time point.
- Venkatesulu et al., 2019 [6]: not given
- Valdès Zayas et al., 2023 [7] (CHUV): Signs of gastrointestinal injury (e.g., body weight loss, diarrhea, and decreased activity) were documented, scored, and used to determine if euthanasia was required as follows: (1) body weight loss of >15%; (2) or a combination of body weight loss of ≥ 15% + immobility; (3) or a combination of body weight loss of ≥ 15% + diarrhea; or a combination of body weight loss + immobility + diarrhea).
- Valdès Zayas et al., 2023 [7] (MD Anderson): Mice were euthanized early (i.e., before the end of the 30-day experimental period) if the following IACUC-approved criteria were met: (1) becoming moribund (exhibiting hunched posture, non-weight-bearing lameness, ruffled fur, laboured breathing) or (2) losing more than 20% of the baseline body weight.
- Hornsey and Alper, 1966 [8]: not given
- Hornsey and Bewley, 1971 [9]: not given

Table S1: Papers considering the endpoint survival fraction.

Papers (particle)	Group	Irradiated region	Tumor bearing?
**Evans et al., 2021 [1] (p)**	Mevion	abdomen	x
**Zhang et al., 2023 [2] (p)**	MGH	abdomen	x
**Zhang et al., 2020 [3] (p)**	MGH	abdomen	x
**Kim et al., 2021 [10] (p)**	UPenn	abdomen	✓
**Velalopoulou et al., 2021 [11] (p)**	UPenn	leg	✓
**Cao et al., 2022 [4] (p)**	UW Medicine	pelvis	x
**Levy et al., 2020 [21] (e)**	Standford	abdomen	x
**Soto et al., 2020 [5] (e)**	Standford	right hemithorax	x
**Venkatesulu et al., 2019 [6] (e)**	MD Anderson	abdomen	x
**Valdés Zayas et al., 2023 [7] (e)**	MD Anderson and CHUV	abdomen	x
**Chabi et al., 2021 (e)**	CHUV	full body	✓
**Favaudon, 2019 [14] (e)**	CHUV	lungs	✓
**Montay-Gruel et al., 2021 [15] (e)**	CHUV	brain	✓
**Leavitt et al., 2024 [16] (e)**	CHUV	full body	✓
**Liljedahl et al., 2022 [17] (e)**	Sweden	brain and flank	✓
**Liljedahl et al., 2024 [18] (e)**	Sweden	brain	✓
**Konradsson et al., 2022 [19] (e)**	Sweden	leg	✓
**Padilla et al., 2024 [20] (e)**	CUIMC	brain	✓
**Hornsey and Alper, 1966 [8] (e)**	London	full body	x
**Hornsey and Bewley, 1971 [9] (e)**	London	full body	x

A2. Crypt cells

The following list will report how the crypt cell analysis was performed in the different groups. The information is extracted from the original papers. In the end, we propose a single, standardized crypt cell regeneration assay that reflects the most common and consistent methodology across all the cited studies.

- Eggold et al., 2022 [21] : Three transverse sections of jejunum were analyzed per mouse. Transverse sections were analyzed if they met the following criteria: (1) a complete jejunal circumference was present, and (2) the mucosa was oriented perpendicular to the long axis of the intestine. Crypts were considered regenerating if they comprised >10 basophilic crypt epithelial cells.

- Levy et al., 2020 [21]: see Eggold et al., 2022 [21]

- Kim et al., 2021 [12]: At 3.5 days post-irradiation, all mice were injected intraperitoneally with 200 mg of 5-ethynyl-20-deoxyuridine (EdU) in phosphate buffer saline 2–3 h before euthanasia. The number of EdU+ cells/crypt and % of regenerated crypts were assessed by counting at least 100 crypts per mouse section. EdU staining was also used to quantify regenerated crypts, defined as crypts containing 5 or more EdU-positive cells with a lumen.

- Diffenderfer et al., 2020 [22]: Two intestine segments OM and DM were harvested. Twenty milligrams per kilogram of 5-ethynyl-20-deoxyuridine (EdU) were injected intraperitoneally 2 to 3 hours before euthanasia staining was detected. EdU cells/crypt were assessed by counting at least 100 crypts per mouse section. EdU staining was also used to quantify the regenerated crypts, where a regenerated crypt contains 5 or more EdU cells with a lumen.

- Ruan et al., 2021 [23]: A modified Swiss roll-based crypt assay was used to quantify the acute crypt damage caused by ionizing radiation. H&E-stained slides were anonymized and scanned using a digital pathology scanner. From each segment, the most severely damaged part, defined by > 3 mm region with the least number of crypts, was chosen from two independent assessments, and the consensus was reached before further counting. The number of crypts from the whole > 3 mm region was counted from each part. Only the crypts with > 10 cells showing no sign of apoptosis were counted as regenerating crypts.

- Valdés Zayas et al., 2023 [7] (CHUV): The jejunum was cleaned, cut open longitudinally, pinned, and fixed in paraformaldehyde for 24 h, washed and then stored in 70% ethanol. The fixed tissues were then processed, embedded in paraffin, sliced in 5-μm sections, and stained with H & E. Surviving crypts were counted manually based on the following criteria: (1) mucosa oriented perpendicular to the long axis of the intestine and (2) the presence of crypts consisted of ≥5 basophilic crypt epithelial cells. Crypts were counted in four randomly selected, identically sized areas of the Swiss-rolled specimens per mouse, and results are reported as the average number of surviving crypts per μm2.

- Valdés Zayas et al., 2023 [7] (Md Anderson): The jejunum from each mouse was collected, fixed in 10% neutral buffered formalin for 24 h, washed once with phosphate-buffered saline (PBS), and stored in 70% ethanol. All tissues were embedded in paraffin, and nine 3-μm thick transverse sections were collected and stained with hematoxylin and eosin (H

& E). Regenerated crypts were counted manually based on the presence of (1) a U-shaped structure with a lumen, (2) a structure along the circumferential edge, (3) a clear, basophilic structure, and (4) multicellularity (at least 10 cells).

- Zhang et al., 2023 [2]: Tissues about 1 cm long were collected from three different intestinal parts (near-end, middle, and far-end) for immunofluorescence staining. The tissue was rinsed with 0.9% saline and embedded with an optimal cutting temperature compound. Ten-micron-thick frozen sections were stained with Ki67 antibody. Only the sections with exceedingly reduced Ki67-positive cells were considered to belong to the irradiated groups.

- Cao et al., 2023 [24]: For EdU (5-ethynyl-2-deoxyuridine) assays, approximately 25mg/kg (0.5mg in a 20g mouse) of 5-ethynyl-20-deoxyuridine (EdU) in phosphate buffer saline (PBS) was injected intraperitoneally into the mice 3-4 hours before euthanasia. The number of EdU+ cells/crypt was assessed by counting at least 100 crypts per mouse section.

Table S2: Papers analyzing the endpoint of crypt cells, time-point of analysis, and way of data reporting. The size of the irradiation field is also reported.

Papers (particle)	Group	Time (days p.i.)	Reported data	Irradiated area (field size cm^2)
**Eggold et al., 2022 [21] (e)**	Standford	4	avg. regenerating crypts per circumference	full intestine (4x4)
**Levy et al., 2020 [12] (e)**	Standford	4	avg. regenerating crypts per circumference	full abdomen (4x3)
**Kim et al., 2021 [10] (p)**	UPenn	3.5	% of remaining crypts, EdU+cells/crypt	full abdomen (2x2)
**Diffenderfer et al., 2020 [22] (p)**	UPenn	3.5	% of remaining crypts, EdU+cells/crypt	full abdomen (1x2)
**Ruan et al., 2021 [23] (e)**	Oxford	3.5	% of remaining crypts	Full abdomen (3.3x3)
**Valdés Zayas et al., 2023 [7] (e)**	Md Anderson	1, 2, 3	no. of surviving crypts	full abdomen (4x4)
**Valdés Zayas et al., 2023 [7] (e)**	CHUV	3.5	avg. regenerating crypts per circumference	full abdomen ($\varnothing 4$)
**Zhang et al., 2023 [2] (p)**	MGH	2	no. of proliferating crypts	60% of abdomen
**Cao et al., 2023 [24] (p)**	UW Medicine	1, 4	no. EdU+cells/crypt	pelvis (1.5x6)

A3. Skin Toxicity

Table S3: List of papers investigating the skin toxicity after irradiation.

Papers (particle)	Group	Irradiated region	Biological model
**Duval et al., 2023 [25] (e)**	Dartmouth	Leg	C57BL/6J
**Tavakkoli et al., 2023 [26] (e)**	Dartmouth	Leg	C57BL/6J
**Soto et al., 2020 [5] (e)**	Standford	Leg	C57BL/6J
**Field and Bewley, 1974 [27] (e)**	London	Foot	F344 rat
**Konradsson et al., 2022 [19] (e)**	Sweden	Leg	F344 rat
**Mascia et al., 2023 [28] (p)**	Cincinnati	Leg	C57BL/6J
**Cunningham et al., 2021 [29] (p)**	Cincinnati	Leg	C57BL/6J
**Sørensen et al., 2022 [30] (p)**	Aarhus	Foot	CDF1
**Singers Sørensen et al., 2022 [31] (p)**	Aarhus	Foot	CDF1
**Iturri, Bertho, et al., 2023 [32] (p)**	Institute Curie	Head	F344 rat
**Rudigkeit et al., 2024 [33] (p)**	Munich	Ear	BALB/c

A4. NOR

This section will summarise how the different groups perform the novel object recognition test.

- **Montay-Gruel et al., 2017 [34]**:
  not clear, reference to Acharya et al., 2011 which does not perform any NOR test.
- **Montay-Gruel et al., 2019 [36]**:
  only a general description of the test was given: "The NOR task involved a sequence of habituation (no objects), familiarisation (two distinct objects), and, lastly, a test phase in which one of the prior objects is switched with a new one."

- **Montay-Gruel et al., 2021 [44]**: reference to Montay-Gruel et al., 2019 [36]

- **Alaghband et al., 2020 [37]**:
  Habituation in empty cage = 10 min/day for 3 days
  Testing - familiarisation = 5 min, 2 objects
  Testing - break = 5 min
  Testing - NOR = 5 min, 2 objects (1 familiar, 1 new)

- **Alaghband et al., 2023 [38]**:
  Habituation in empty cage = 5 min for 1 day
  Testing - familiarisation = 5 min, 2 objects
  Testing - break = 5 min
  Testing - NOR = 5 min, 2 objects (1 familiar, 1 new)

- **Allen et al., 2023 [39]**: reference to Alaghband et al., 2020 [37]

- **Almeida et al., 2023 [40]**: reference to Montay-Gruel et al., 2017 [34]

- **Iturri, Bertho, Lamirault, Brisebard, et al., 2023 [32]**:
  Habituation in empty cage = 3 min for 2 times with 3h break for 1 day
  Testing - familiarisation = 5 min, 2 objects
  Testing - break = 3h
  Testing - NOR = 5 min, 2 objects (1 familiar, 1 new)

- **Iturri, Bertho, Lamirault, Juchaux, et al., 2023 [41]**:
  see Iturri, Bertho, Lamirault, Brisebard, et al., 2023

- **Simmons et al., 2019 [42]**:
  Habituation in empty cage = 10 min for 1 day
  Testing - familiarisation = 10 min, 2 objects
  Testing - break = 1h
  Testing - NOR = 10 min, 2 objects (1 familiar, 1 new)

- **Williams et al., 2022 [43]:**
  Habituation in empty cage = ?
  Testing - familiarisation = 10 min, 4 objects
  Testing - break = 1h
  Testing - NOR = 10 min, 2 objects (3 familiar, 1 new)

Table S4: Papers performing the novel object recognition test. The metric for evaluating the NOR varies between the papers: the discrimination index (DI) is calculated by normalizing the normalized time spent exploring the familiar object from the normalized time spent exploring the novel object by the total exploration time. The recognition ratio (RR) is the ratio between the time spent investigating the novel object and the total exploration time. RR* is a modified RR in which the time spent investigating the novel object is divided by 30 seconds.

Papers (particle)	Group	Metric	Time (months p.i.)	Biological model
**Montay-Gruel et al., 2017 [34] (e)**	CHUV	RR	2, 6	C57BL/6J
**Montay-Gruel et al., 2019 [36] (e)**	CHUV	DI	2	C57BL/6J
**Montay-Gruel et al., 2021 [44] (e)**	CHUV	DI	1	NU/NU nude mice
**Alaghband et al., 2020 [37] (e)**	CHUV	DI	4	C57BL/6J
**Alaghband et al., 2023 [38] (e)**	CHUV	DI	4	C57BL/6J
**Allen et al., 2023 [39] (e)**	CHUV	DI	4	C57BL/6J
**Almeida et al., 2023 [40] (e,p)**	CHUV, PSI	RR	2	C57BL/6J
**Iturri et al., 2023 [32] (p)**	Institute Curie	DI	1, 3, 6	F344 rat
**Iturri, Bertho, et al., 2023 [41] (p)**	Institute Curie	DI	3	F344 rat
**Simmons et al., 2019 [42] (e)**	Standford	DI	2.5	C57BL/6J
**Williams et al., 2022 [43] (p)**	Cincinnati	RR*	2.5	Sprague Dawley CD rat

A5. Suggestions for standardisation

Standardizing reporting and analysis of common endpoints will be an important step towards achieving consensus in the description of the FLASH effect. Ideally it should be done within international societies; here, we make a first proposal in this direction, based on our observations on the literature presented above.

Table S5: Recommendations for future Preclinical FLASH Studies based on the most commonly used modalities and definitions for the analysed endpoints.

ENDPOINT	RECOMMENDATIONS on the METHODOLOGY	METRIC	TIMING of ANALYSIS
**Survival (after abdominal irradiation)**	Clearly report the euthanasia criteria used in the study	Survival probability and time-to-event data	Record up to 20 days post-irradiation
**Crypt Cells**	• Define clearly what a regenerating (surviving) crypt is. Examples from the literature can be found in A2<br>• Consider automated techniques for crypt counting [86].<br>• As various methods exist for counting regenerative crypt cells, including the transverse-section (cross-section) and Swiss-roll approaches, we recommend clearly reporting the methodology used. Standard protocols (see [47-49]) provide a proven framework for consistent preparation and analysis.	Report both raw and normalized metrics (crypts per section and crypts/mm^2 or crypts/mm) together with the measured circumference length (mm) or area (mm^2), allowing readers to compare across methods. Report the irradiated area.	3.5-4 days post-irradiation
**Skin toxicity**	• We recommend focusing on moist desquamation. Healing timing can be assessed, but it should not be the primary endpoint.<br>• Proper skin preparation before irradiation is crucial [50]. Avoid depilation, tape, or any irritants that could affect the skin or skew results.	Percentage of mice reaching moist desquamation.<br>If multiple skin toxicity levels are of interest, consider a multi-level grading scheme [46].	From irradiation until the end of the healing period

**NOR**	Several ways exist to perform the NOR test; we highlight the standardized protocol proposed in Leger, M., et al. (2013) [45]. This protocol facilitates comparability across studies and can be tailored to the experimenter's specific needs.	Use the discrimination index or a convertible metric.<br><br>Consider normalizing for the control group.	At least 2-4 months post-irradiation

Supplementary B: Endpoint analysis

B1. Survival Ratio

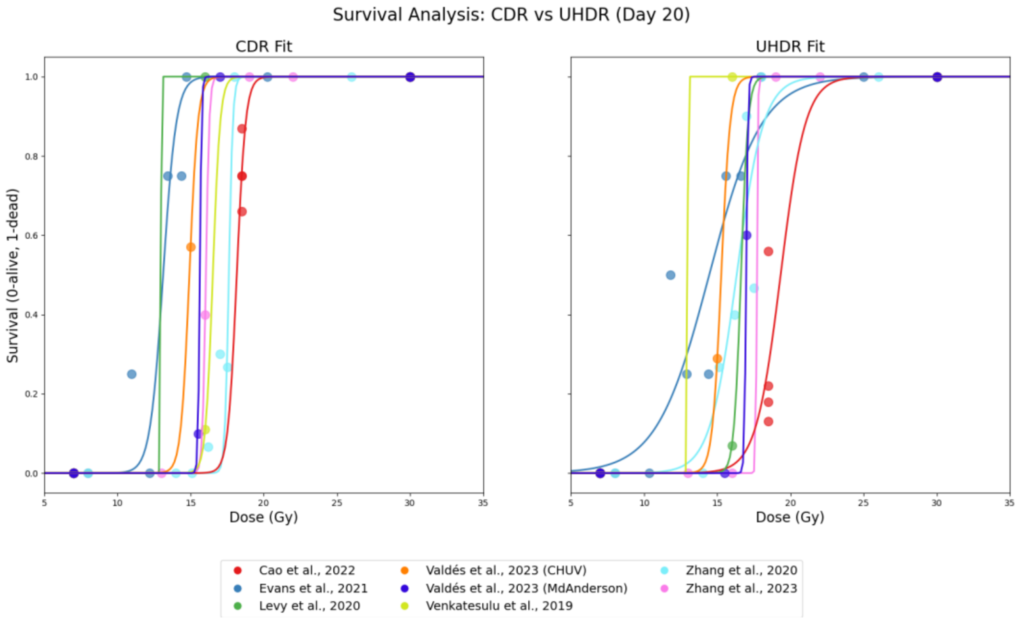

Figure S1: Survival rate at day 20 as a function of dose for CDR and UHDR irradiated mice. A weighted logistic fit (1/(1 + e^{(a-x)/b}) over all the data points of each group is performed in both cases. The fitting parameters (a,b) with their 95\% confidence intervals are reported in the table below. The compared papers are listed in Table S1.

Table S6: (a,b) parameters of the weighted logistic fit (1/(1 + e^{(a-x)/b}) presented in Figure S1 (fit performed adding additional data at low dose and high dose)

Study	Condition	a (dose@50%) ± CI	b (slope) ± CI
Evans et al., 2021 [1]	CDR	13.126 ± 0.419	0.445 ± 0.333
	UHDR	14.448 ± 0.983	1.797 ± 0.943
Zhang et al., 2020 [2]	CDR	16.047 ± 0.004	0.116 ± 0.009
	UHDR	17.685 ± 5.0×10^{49}	0.036 ± 1.1×10^{39}
Zhang et al., 2023 [3]	CDR	17.618 ± 0.149	0.135 ± 0.134
	UHDR	16.324 ± 0.588	0.919 ± 0.565
Cao et al., 2022 [4]	CDR	18.237 ± 2.2×10^{17}	0.231 ± 1.9×10^{17}
	UHDR	19.317 ± 1399.730	0.832 ± 1425.411
Levy et al., 2020 [21]	CDR	12.920 ± 1.0×10^{49}	0.016 ± 1.0×10^{49}

	UHDR	16.64 ± 25.978	0.247 ± 10.843
Venkatesulu et al., 2019 [6]	CDR	16.687 ± 0.029	0.329 ± 0.014
	UHDR	Fit failed	Fit failed
Valdés Zayas et al., 2023 (CHUV) [7]	CDR	14.919 ± 0.003	0.288 ± 0.012
	UHDR	15.277 ± 0.012	0.309 ± 0.014
Valdés Zayas et al., 2023 (MD) [7]	CDR	15.630 ± 0.001	0.059 ± 0.000
	UHDR	16.975 ± 0.001	0.060 ± 0.002

B2. Crypt cells

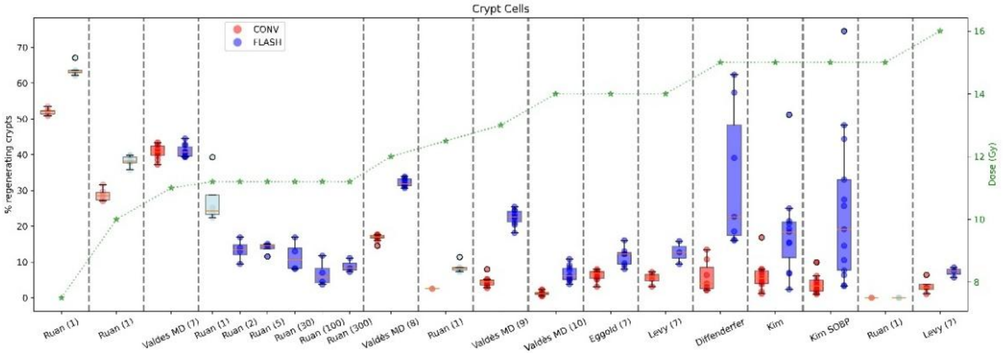

Figure S2: Crypt cell analysis with all published data non-weighted for the irradiated area.

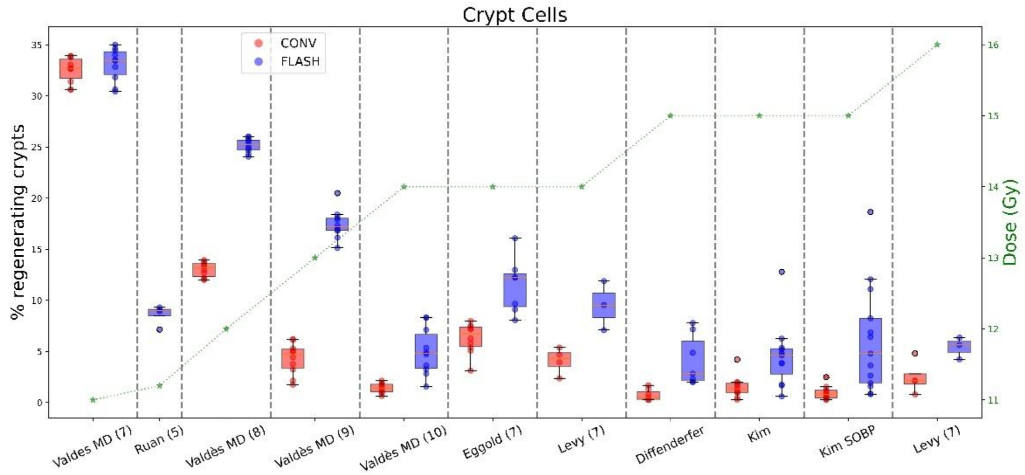

Figure S3.1: control 130 crypt cells/circumference

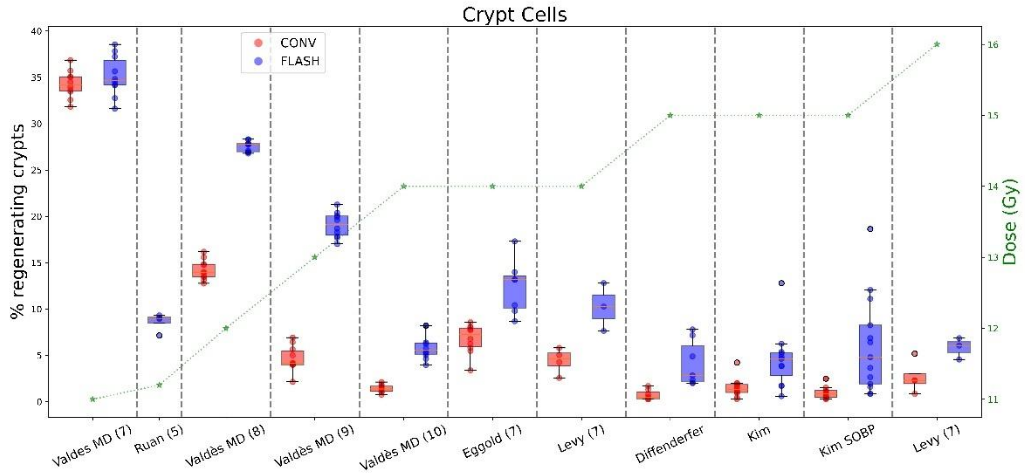

Figure S3.2: control 140 crypt cells/circumference

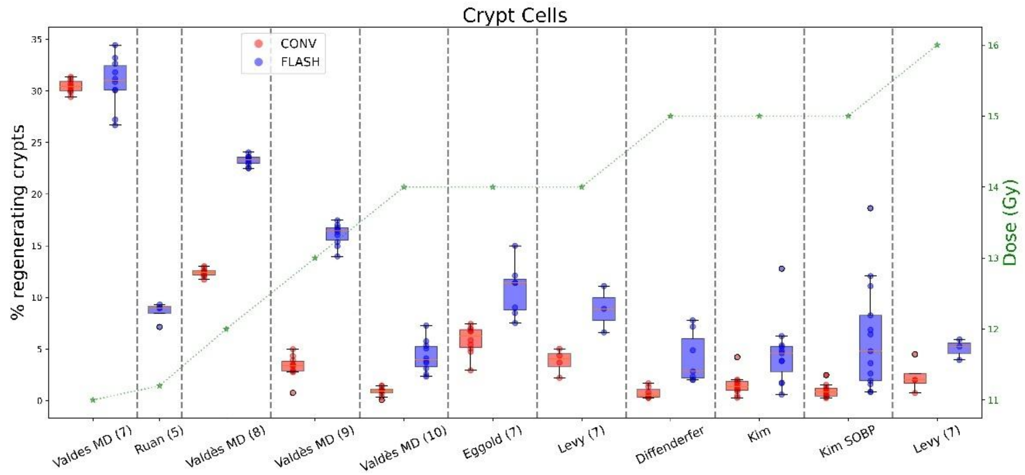

Figure S3.2: control 150 crypt cells/circumference

Figure S3: Variation of the results using different baseline number of crypt per circumference.

B3. Skin Toxicity

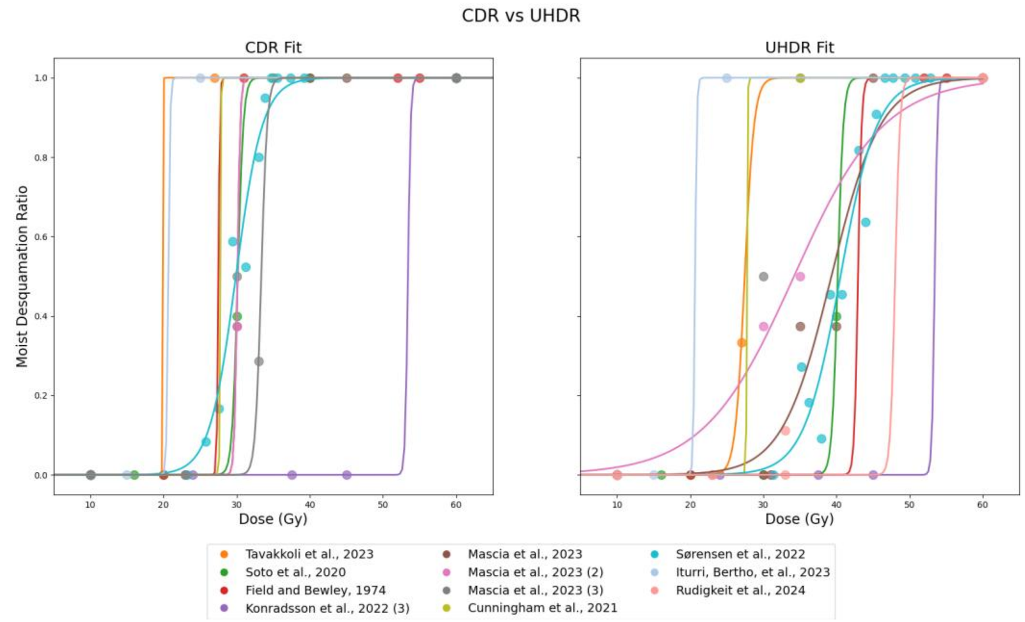

Figure S4: Moist desquamation ratio as a function of dose for CDR and UHDR irradiated mice. A weighted logistic fit (1/(1 + e^{(a-x)/b}) over all the data points of each group is performed in both cases. The fitting parameters (a,b) with their 95\% confidence intervals are reported in the table below. The compared papers are listed in Table S3. Multiple deliveries are indicated in brackets.

Table S7: (a,b) parameters of the weighted logistic fit (1/(1 + e^{(a-x)/b}) presented in Figure S5 (fit performed adding additional data at low dose and high dose)

STUDY	CONDITION	A (DOSE@50%) ± CI	B (SLOPE) ± CI
Tavakkoli et al., 2023 [26]	CDR	20.825 ± ∞	0.029 ± ∞
	UHDR	27.447 ± 0.019	0.644 ± 0.028
Soto et al., 2020 [5]	CDR	30.154 ± 0.011	0.379 ± 0.027
	UHDR	40.160 ± 0.005	0.393 ± 0.012
Field and Bewley, 1974 [27]	CDR	27.506 ± 3.919e+16	0.096 ± 8.543e+14
	UHDR	42.966 ± 1.735e+17	0.259 ± 3.832e+15
Konradsson et al., 2022 (3) [19]	CDR	53.492 ± 2.841e+21	0.182 ± 6.210e+19
	UHDR	53.492 ± 2.841e+21	0.182 ± 6.210e+19
Mascia et al., 2023 [28]	CDR	30.100 ± 2.598e+30	0.196 ± 5.086e+30
	UHDR	39.299 ± 2.383	3.357 ± 2.251
Mascia et al., 2023 (2) [28]	CDR	30.100 ± 0.000	0.196 ± 0.000

	UHDR	34.185 ± 1.289	6.150 ± 1.907
Mascia et al., 2023 (3) [28]	CDR	30.000 ± 0.000	0.226 ± 9.893e+25
	UHDR	30.000 ± 0.000	0.226 ± 9.893e+25
Cunningham et al., 2021 [29]	CDR	25.148 ± ∞	0.274 ± ∞
	UHDR	25.148 ± ∞	0.274 ± ∞
Sørensen et al., 2022 [31]	CDR	29.903 ± 0.592	1.823 ± 0.491
	UHDR	40.545 ± 0.827	2.518 ± 0.710
Iturri, Bertho, et al., 2023 [32]	CDR	20.779 ± 3.685e+19	0.120 ± 7.827e+17
	UHDR	20.779 ± 3.685e+19	0.120 ± 7.827e+17
Rudigkeit et al., 2024 [33]	CDR	33.369 ± 0.015	0.403 ± 0.016
	UHDR	48.229 ± 1.061e+17	0.331 ± 2.359e+15

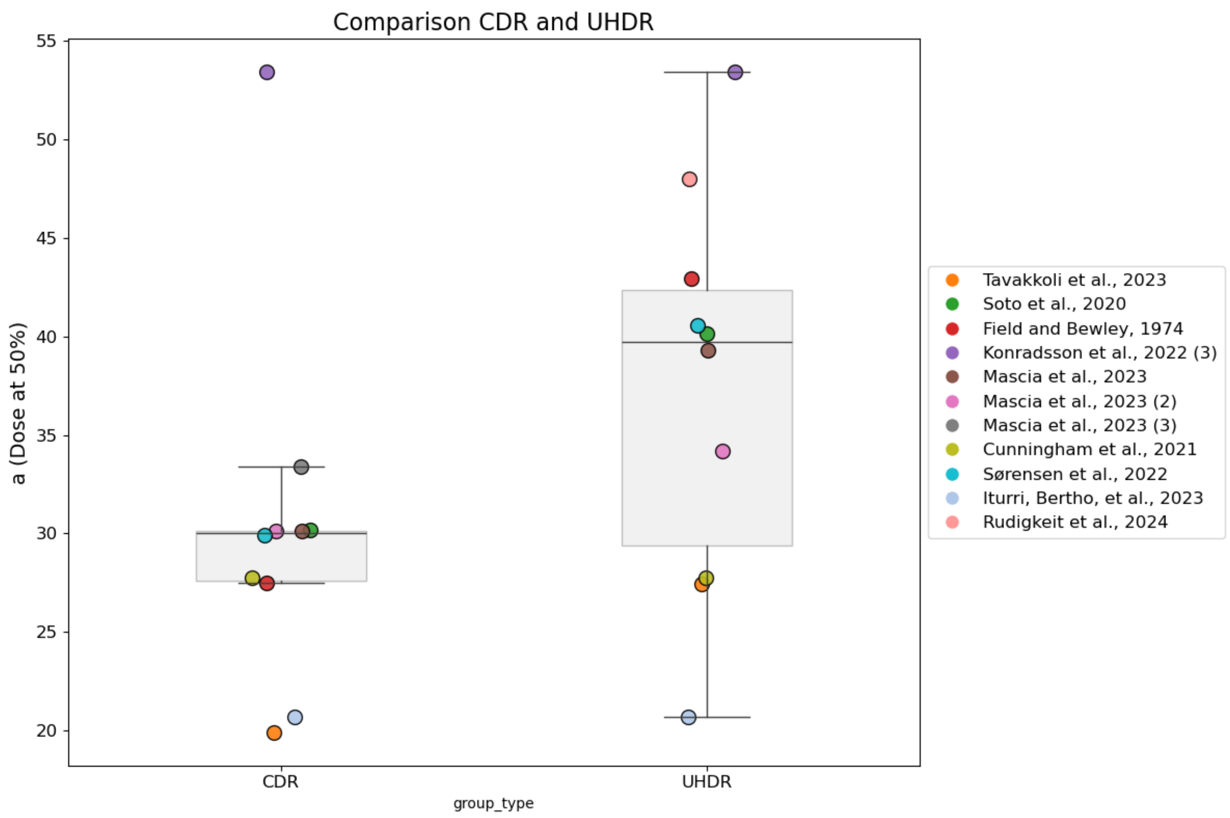

Figure S5: a value of the logistic fit grouped in CRD and UHDR.

B4. NOR test

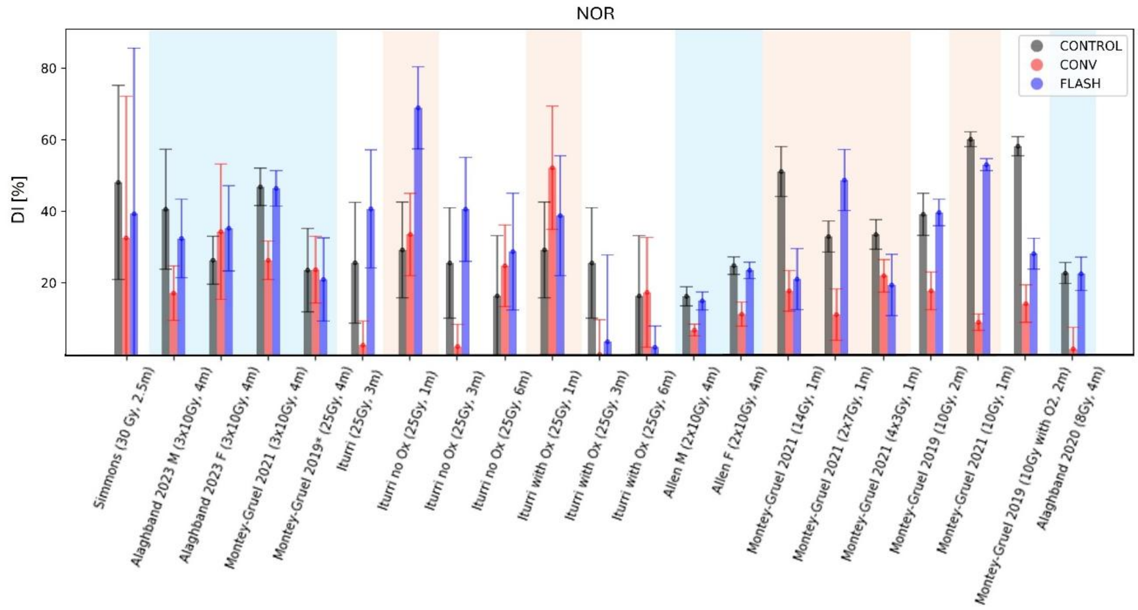

Figure S6: NOR non-normalized to motivate the choice of normalizing the data for the control group given the large variability in the magnitude of DI already in the control group. The different background colors highlight the differences months after irradiation at which the study was performed. The data are expressed as median value +/- SEM.

Supplementary C: statistical analysis

C1. Survival rate

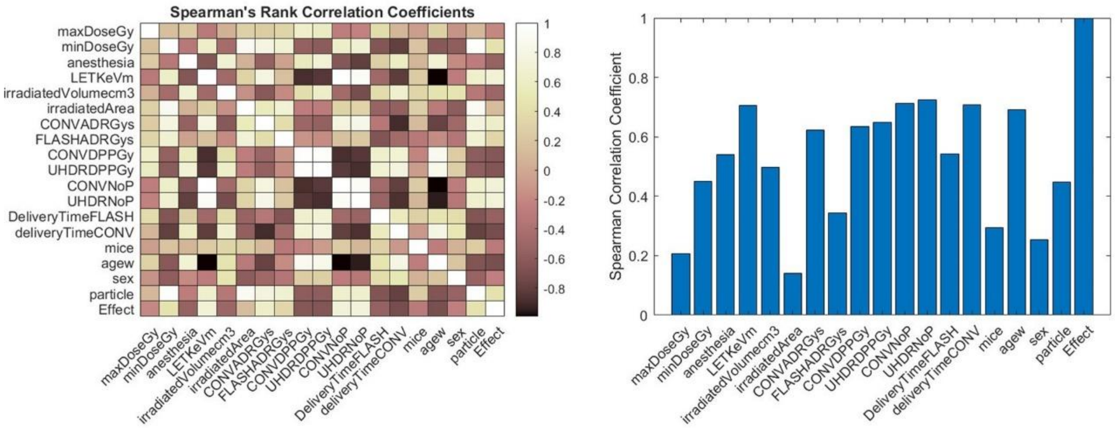

Figure S7: Survival rate analysis. (Left) Spearman's Rank Correlation Coefficient matrix. A coefficient of 1 indicates a perfect monotonic relationship (increasing), -1 indicates a perfect negative monotonic relationship (decreasing), and 0 indicates no monotonic relationship. (Right) Focus on the absolute value of the Spearman's Rank Correlation Coefficient for the variable "Effect".

Table S8: *The resulting model of survival effect analysis is given by Logit(Effect) ~ 1 + Anesthesia + Irradiated Volume + Rodents sex + Max Dose + Delivery time (UHDR) + Average Dose Rate (UHDR), has a pValue **** and a McFadden's R-squared value of 1. AUC = 1. Only uncorrelated parameters are included.*

Variable	Estimate	p Value	BS selection freq./1000
**Anesthesia**	-2.2912	**	872
**Irradiated Volume**	2.5007	**	953
**Rodents sex**	-1.7963	**	872
**Rodents strain**	/	/	62
**Max Dose**	0.61632	ns	802
**Min Dose**	/	/	53
**Delivery time (UHDR)**	-2.3772	**	935
**Average Dose Rate (UHDR)**	-1.8825	*	839
**Number of Pulses (UHDR)**	/	/	145

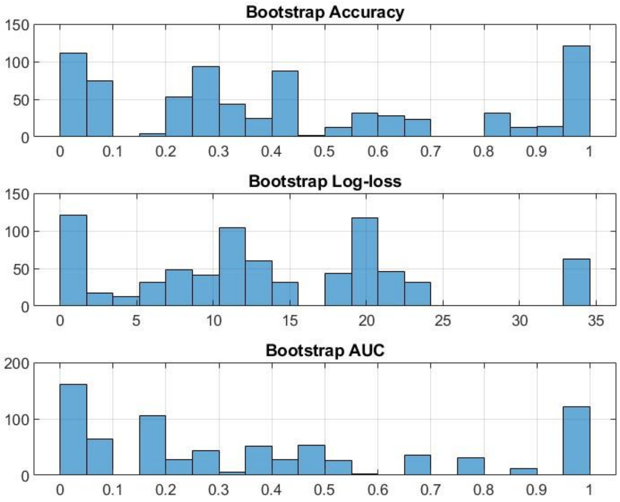

Figure S8: Survival rate analysis. Model performance after Bootstrapping. The following performance metrics are computed across 1000 bootstrap samples: Accuracy: 0.419 (95% CI: 0.000 – 1.000), Log-loss: 14.146 (95% CI: 0.000 – 34.539), AUC: 0.358 (95% CI: 0.000 – 1.000). After the LOGO-CV test, we obtain the following performance metrics: an Accuracy of 0.32, a Log-loss of 14.76, and an AUC of 0.27

C2. Moist desquamation

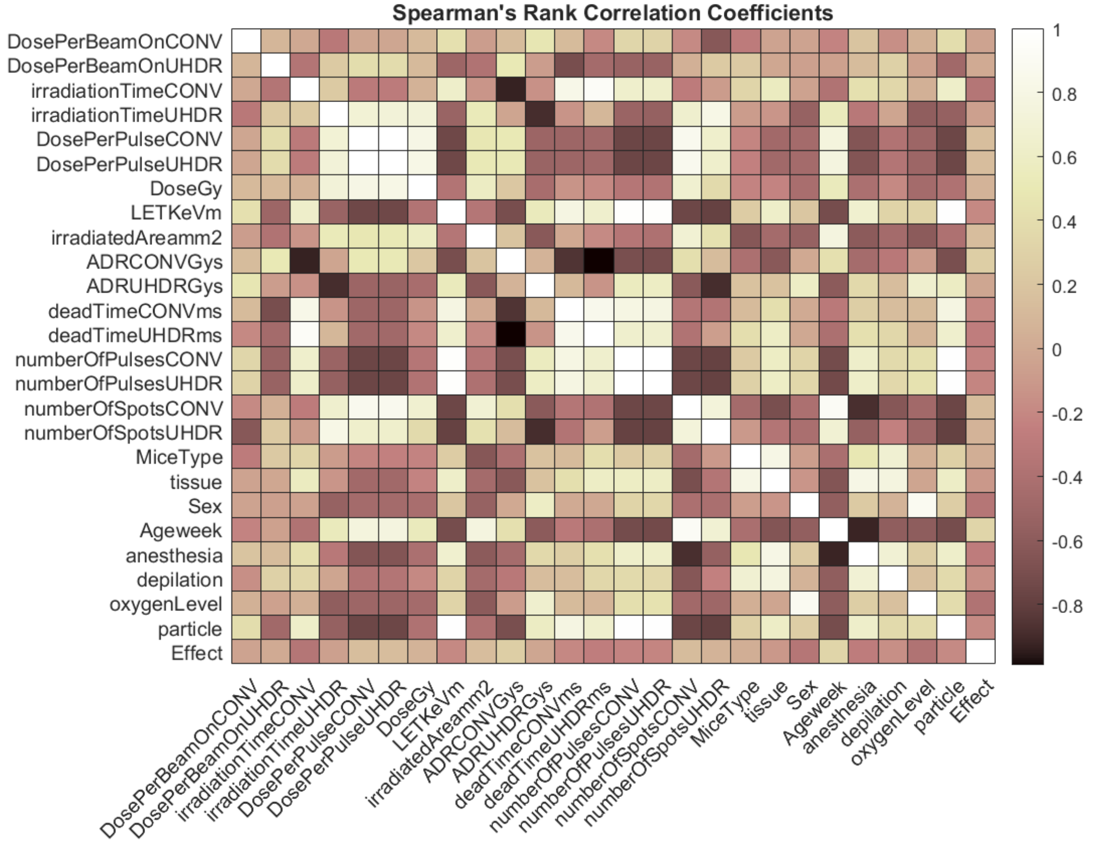

Figure S9: Moist desquamation analysis. Spearman's Rank Correlation Coefficient matrix. A coefficient of 1 indicates a perfect monotonic relationship (increasing), -1 indicates a perfect negative monotonic relationship (decreasing), and 0 indicates no monotonic relationship.

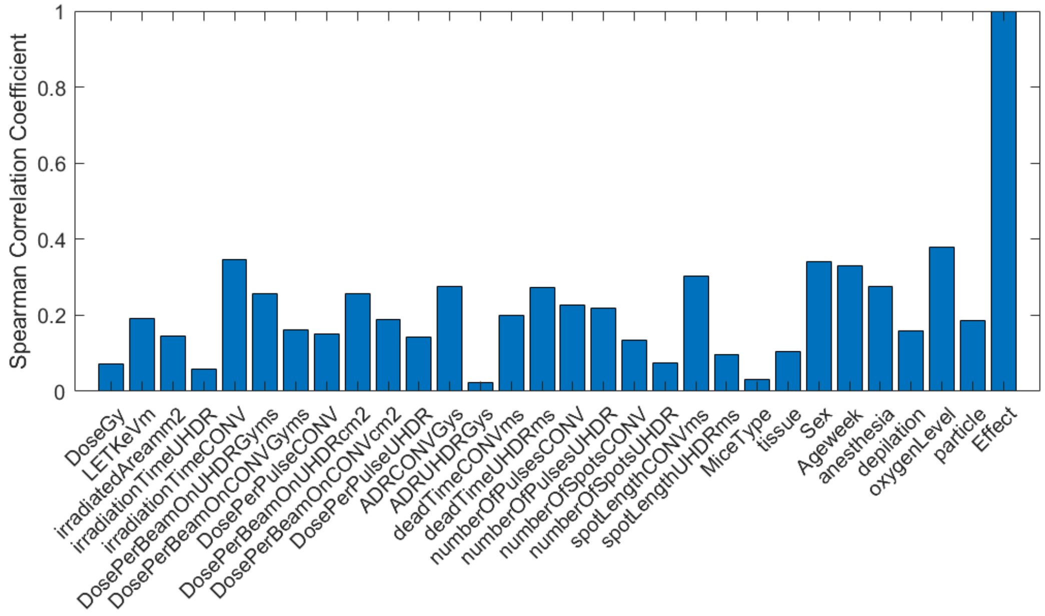

Figure S10: Moist desquamation analysis. Focus on the absolute value of the Spearman's Rank Correlation Coefficient for the variable "Effect".

Table S9: *The resulting model of moist desquamation analysis is given by Logit(Effect) ~ 1 + Dose per pulse or spot length per area (CONV) + Dose Per Pulse (UHDR) + Irradiation Time (CONV) + Spot Length (UHDR) + Irradiated tissue + Rodents Age + Depilation + Oxygen Level , has a pValue **** and a McFadden's R-squared value of 0.8. AUC = 1. Only uncorrelated parameters are included.*

Variable	Estimate	p Value	BS selection freq./1000
**Dose per pulse or spot length per area (CONV)**	-33.111	****	1000
**Dose Per Pulse (UHDR)**	27.606	****	999
**Irradiation Time (CONV)**	6.5856	*	566
**Number of Pulses (UHDR)**	540.98	****	1000
**Number of Spots (UHDR)**	/	/	381
**Spot Length (UHDR)**	-536.7	****	1000
**Oxygen Level**	-2358.1	ns	1000
**Irradiated tissue**	-1473.1	****	1000
**Rodents Age**	-1762.8	****	1000
**Depilation**	-287.5	****	999

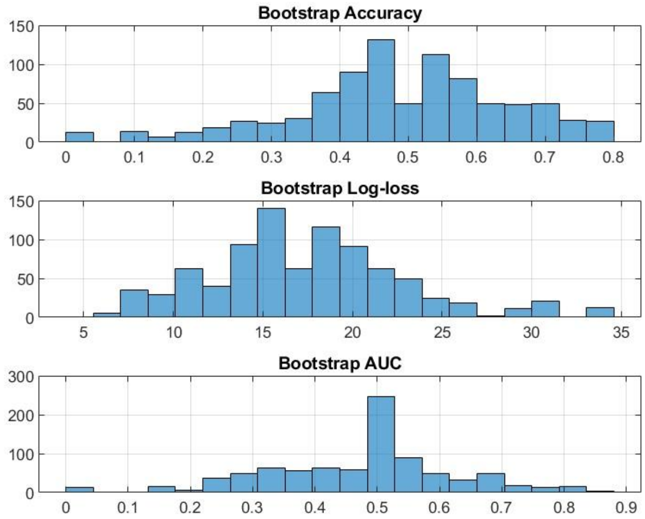

Figure S11: Moist desquamation analysis. Model performance after Bootstrapping. The following performance metrics are computed across 1000 bootstrap samples: Accuracy: 0.487 (95% CI: 0.105 – 0.775), Log-loss: 17.451 (95% CI: 7.509 – 30.904), AUC: 0.472 (95% CI: 0.147 – 0.789). After the LOGO-CV test, we obtain the following performance metrics: an Accuracy of 0.46, a Log-loss of 18.48, and an AUC of 0.47.

References

[1] Evans T, Cooley J, Wagner M, Yu T, Zwart T. Demonstration of the FLASH effect within the spread-out Bragg peak after abdominal irradiation of mice. Int J Part Ther. 2021;8:68-75. https://doi.org/10.14338/IJPT-20-00095.1

[2] Zhang Q, Gerweck LE, Cascio E, et al. Absence of tissue-sparing effects in partial proton FLASH irradiation in murine intestine. Cancers. 2023;15:2269. https://doi.org/10.3390/cancers15082269

[3] Zhang Q, Cascio E, Li C, et al. FLASH investigations using protons: Design of delivery system, preclinical setup and confirmation of FLASH effect with protons in animal systems. Radiat Res. 2020;194:656-664. https://doi.org/10.1667/RADE-20-00068.1

[4] Cao N, Erickson DPJ, Tan S, et al. FLASH vs. conventional dose rate proton radiation in mouse abdomen. Int J Radiat Oncol Biol Phys. 2022;114:e520. https://doi.org/10.1016/j.ijrobp.2022.07.2109

[5] Soto LA, Casey KM, Wang J, et al. FLASH irradiation results in reduced severe skin toxicity compared to conventional-dose-rate irradiation. Radiat Res. 2020;194:618-624. https://doi.org/10.1667/RADE-20-00090.1

[6] Venkatesulu BP, Sharma A, Pollard-Larkin JM, et al. Ultra high dose rate (35 Gy/sec) radiation does not spare the normal tissue in cardiac and splenic models of lymphopenia and gastrointestinal syndrome. Sci Rep. 2019;9:17180. https://doi.org/10.1038/s41598-019-53562-y

[7] Zayas AV, Ramos G, Podesta M, et al. Independent reproduction of the FLASH effect on the gastrointestinal tract: A multi-institutional comparative study. Cancers. 2023;15:2121. https://doi.org/10.3390/cancers15072121

[8] Hornsey S, Alper T. Unexpected dose-rate effect in the killing of mice by radiation. Nature. 1966;210:212-213. https://doi.org/10.1038/210212a0

[9] Hornsey S, Bewley DK. Hypoxia in mouse intestine induced by electron irradiation at high dose-rates. Int J Radiat Biol Relat Stud Phys Chem Med. 1971;19:479-483. https://doi.org/10.1080/09553007114550611

[10] Kim MM, Verginadis II, Goia D, et al. Comparison of FLASH proton entrance and the spread-out Bragg peak dose regions in the sparing of mouse intestinal crypts and in a pancreatic tumor model. Cancers. 2021;13:4244. https://doi.org/10.3390/cancers13164244

[11] Velalopoulou A, Karagounis IV, Cramer GM, et al. FLASH proton radiotherapy spares normal epithelial and mesenchymal tissues while preserving sarcoma response. Cancer Res. 2021;81:4808-4821. https://doi.org/10.1158/0008-5472.CAN-21-1500

[12] Levy K, Natarajan S, Wang J, et al. Abdominal FLASH irradiation reduces radiation-induced gastrointestinal toxicity for the treatment of ovarian cancer in mice. Sci Rep. 2020;10:21600. https://doi.org/10.1038/s41598-020-78017-7

[13] Chabi S, To THV, Leavitt R, et al. Ultra-high-dose-rate FLASH and conventional-dose-rate irradiation differentially affect human acute lymphoblastic leukemia and normal hematopoiesis. Int J Radiat Oncol Biol Phys. 2021;109:819-829. https://doi.org/10.1016/j.ijrobp.2020.10.012

[14] Favaudon V, Lentz JM, Heinrich S, et al. FLASH radiotherapy at very high dose-rate: A brief account of the current situation. Cancer Radiother. 2019;23:674-676. https://doi.org/10.1016/j.canrad.2019.06.003

[15] Montay-Gruel P, Acharya MM, Gonçalves Jorge P, et al. Hypofractionated FLASH-RT as an effective treatment against glioblastoma that reduces neurocognitive side effects in mice. Clin Cancer Res. 2021;27:775-784. https://doi.org/10.1158/1078-0432.CCR-20-0894

[16] Leavitt RJ, Almeida A, Grilj V, et al. Acute hypoxia does not alter tumor sensitivity to FLASH radiotherapy. Int J Radiat Oncol Biol Phys. 2024;120:1373-1381. https://doi.org/10.1016/j.ijrobp.2024.02.015

[17] Liljedahl E, Konradsson E, Gustafsson E, et al. Long-term anti-tumor effects following both conventional radiotherapy and FLASH in fully immunocompetent animals with glioblastoma. Sci Rep. 2022;12:12285. https://doi.org/10.1038/s41598-022-16612-6

[18] Liljedahl E, Konradsson E, Linderfalk K, et al. Comparable survival in rats with intracranial glioblastoma irradiated with single-fraction conventional radiotherapy or FLASH radiotherapy. Front Oncol. 2024;13:1309174. https://doi.org/10.3389/fonc.2023.1309174

[19] Konradsson E, Liljedahl E, Gustafsson E, et al. Comparable long-term tumor control for hypofractionated FLASH versus conventional radiation therapy in an immunocompetent rat glioma model. Adv Radiat Oncol. 2022;7:101011. https://doi.org/10.1016/j.adro.2022.101011

[20] Padilla O, Minns HE, Wei HJ, et al. Immune response following FLASH and conventional radiation in diffuse midline glioma. Int J Radiat Oncol Biol Phys. 2024;119:1243-1254. https://doi.org/10.1016/j.ijrobp.2024.01.219

[21] Eggold JT, Chow S, Melemenidis S, et al. Abdominopelvic FLASH irradiation improves PD-1 immune checkpoint inhibition in preclinical models of ovarian cancer. Mol Cancer Ther. 2022;21:371-381. https://doi.org/10.1158/1535-7163.MCT-21-0358

[22] Diffenderfer ES, Verginadis II, Kim MM, et al. Design, implementation, and in vivo validation of a novel proton FLASH radiation therapy system. Int J Radiat Oncol Biol Phys. 2020;106:440-448. https://doi.org/10.1016/j.ijrobp.2019.10.049

[23] Ruan JL, Lee C, Wouters S, et al. Irradiation at ultra-high (FLASH) dose rates reduces acute normal tissue toxicity in the mouse gastrointestinal system. Int J Radiat Oncol Biol Phys. 2021;111:1250-1261. https://doi.org/10.1016/j.ijrobp.2021.08.004

[24] Cao N, Erickson DPJ, Ford EC, et al. Preclinical ultra-high dose rate FLASH proton radiotherapy system for small animal studies. Adv Radiat Oncol. 2023;9:101425. https://doi.org/10.1016/j.adro.2023.101425

[25] Duval KEA, Aulwes E, Zhang R, et al. Comparison of tumor control and skin damage in a mouse model after ultra-high dose rate irradiation and conventional irradiation. Radiat Res. 2023;200:223-231. https://doi.org/10.1667/RADE-23-00057

[26] Tavakkoli AD, Clark MA, Kheirollah A, et al. Anesthetic oxygen use and sex are critical factors in the FLASH sparing effect. bioRxiv. 2023. https://doi.org/10.1101/2023.11.04.565626

[27] Field SB, Bewley DK. Effects of dose-rate on the radiation response of rat skin. Int J Radiat Biol Relat Stud Phys Chem Med. 1974;26:259-267. https://doi.org/10.1080/09553007414551221

[28] Mascia A, Daugherty EC, Zhang Y, et al. Impact of multiple beams on the FLASH effect in soft tissue and skin in mice. Int J Radiat Oncol Biol Phys. 2023;118:253-261. https://doi.org/10.1016/j.ijrobp.2023.07.024

[29] Cunningham S, McCauley S, Vairamani K, et al. FLASH proton pencil beam scanning irradiation minimizes radiation-induced leg contracture and skin toxicity in mice. Cancers. 2021;13:1012. https://doi.org/10.3390/cancers13051012

[30] Sørensen BS, Sitarz MK, Ankjærgaard C, et al. Pencil beam scanning proton FLASH maintains tumor control while normal tissue damage is reduced in a mouse model. Radiother Oncol. 2022;175:178-184. https://doi.org/10.1016/j.radonc.2022.05.014

[31] Sørensen BS, Sitarz MK, Ankjærgaard C, et al. In vivo validation and tissue sparing factor for acute damage of pencil beam scanning proton FLASH. Radiother Oncol. 2022;167:109-115. https://doi.org/10.1016/j.radonc.2021.12.022

[32] Iturri L, Bertho A, Lamirault C, et al. Oxygen supplementation in anesthesia can block FLASH effect and anti-tumor immunity in conventional proton therapy. Commun Med. 2023;3:176. https://doi.org/10.1038/s43856-023-00411-9

[33] Rudigkeit S, Schmid TE, Dombrowsky AC, et al. Proton-FLASH: Effects of ultra-high dose rate irradiation on an in-vivo mouse ear model. Sci Rep. 2024;14:1418. https://doi.org/10.1038/s41598-024-51951-6

[34] Montay-Gruel P, Petersson K, Jaccard M, et al. Irradiation in a flash: Unique sparing of memory in mice after whole brain irradiation with dose rates above 100 Gy/s. Radiother Oncol. 2017;124:365-369. https://doi.org/10.1016/j.radonc.2017.05.003

[35] Acharya MM, Christie LA, Lan ML, et al. Human neural stem cell transplantation ameliorates radiation-induced cognitive dysfunction. Cancer Res. 2011;71:4834-4845. https://doi.org/10.1158/0008-5472.CAN-11-0027

[36] Montay-Gruel P, Acharya MM, Petersson K, et al. Long-term neurocognitive benefits of FLASH radiotherapy driven by reduced reactive oxygen species. Proc Natl Acad Sci USA. 2019;116:10943-10951. https://doi.org/10.1073/pnas.1901777116

[37] Alaghband Y, Cheeks SN, Allen BD, et al. Neuroprotection of radiosensitive juvenile mice by ultra-high dose rate FLASH irradiation. Cancers. 2020;12:1671. https://doi.org/10.3390/cancers12061671

[38] Alaghband Y, Allen BD, Kramar EA, et al. Uncovering the protective neurologic mechanisms of hypofractionated FLASH radiotherapy. Cancer Res Commun. 2023;3:725-737. https://doi.org/10.1158/2767-9764.CRC-23-0117

[39] Allen BD, Alaghband Y, Kramar EA, et al. Elucidating the neurological mechanism of the FLASH effect in juvenile mice exposed to hypofractionated radiotherapy. Neuro Oncol. 2023;25:927-939. https://doi.org/10.1093/neuonc/noac248

[40] Almeida A, Togno M, Ballesteros-Zebadua P, et al. Dosimetric and biologic intercomparison between electron and proton FLASH beams. Radiother Oncol. 2023;187:109953. https://doi.org/10.1016/j.radonc.2023.109953

[41] Iturri L, Bertho A, Lamirault C, et al. Proton FLASH radiation therapy and immune infiltration: Evaluation in an orthotopic glioma rat model. Int J Radiat Oncol Biol Phys. 2023;116:655-665. https://doi.org/10.1016/j.ijrobp.2023.01.018

[42] Simmons DA, Lartey FM, Schüler E, et al. Reduced cognitive deficits after FLASH irradiation of whole mouse brain are associated with less hippocampal dendritic spine loss and neuroinflammation. Radiother Oncol. 2019;139:4-10. https://doi.org/10.1016/j.radonc.2019.06.006

[43] Williams MT, Sugrue M, Vlasakova E, et al. Cognitive and behavioral effects of whole brain conventional or high dose rate (FLASH) proton irradiation in a neonatal Sprague Dawley rat model. PLoS One. 2022;17:e0274007. https://doi.org/10.1371/journal.pone.0274007

[44] Montay-Gruel P, Acharya MM, Gonçalves Jorge P, et al. Hypofractionated FLASH-RT as an effective treatment against glioblastoma that reduces neurocognitive side effects in mice. Clin Cancer Res. 2021;27:775-784. https://doi.org/10.1158/1078-0432.CCR-20-0894

[45] Leger M, Quiedeville A, Bouet V, et al. Object recognition test in mice. Nat Protoc. 2013;8:2531-2537. https://doi.org/10.1038/nprot.2013.155

[46] Maase H. Effect of cancer chemotherapeutic drugs on the radiation-induced skin reactions in mouse feet. Br J Radiol. 1984;57:697-707. https://doi.org/10.1259/0007-1285-57-680-697

[47] Kuo HC, Kirsch DG. Histological assessment of intestinal injury by ionizing radiation. Methods Cell Biol. 2023;180:147-175. https://doi.org/10.1016/bs.mcb.2023.03.001

[48] Williams JM, Duckworth CA, Burkitt MD, et al. Intestinal preparation techniques for histological analysis in the mouse. Curr Protoc Mouse Biol. 2016;6:148-168. https://doi.org/10.1002/cpmo.2

[49] Tustison KS, Eaves CJ. Assessment of intestinal stem cell survival using the microcolony formation assay. In: Mitry S, ed. Methods in Molecular Medicine. Vol 50. Hematopoietic Stem Cell Protocols. Humana Press; 1998:267-273. https://doi.org/10.1385/1-59259-084-5:267

[50] Baikalov A, Peng Y, Liu K, Hadsell M, Schüler E, Gladstone DJ. Modernizing histopathological analysis: A fully automated workflow for the digital image analysis of the intestinal microcolony survival assay. bioRxiv. 2024. https://doi.org/10.1101/2024.12.09.627578
